# MEETING THE CHALLENGE: NEURAL RESPONSE ASSOCIATED WITH MINIMAL DUAL-TASK COST IN AGING

**DOI:** 10.1093/geroni/igae098.3671

**Published:** 2024-12-31

**Authors:** Claudia Schneider, Sophie Molholm, John Foxe, Johanna Wagner, Pierfilippo De Sanctis

**Affiliations:** Albert Einstein College of Medicine, Bronx, New York, United States; Albert Einstein College of Medicine, Bronx, New York, United States; University of Rochester Medical Center, Rochester, New York, United States; Zander Labs, Cottbus, Brandenburg, Germany; Albert Einstein College of Medicine, Bronx, New York, United States

## Abstract

Walking while engaged in a cognitively challenging task is a common daily activity that becomes harder as we grow older. The cost associated with performing two tasks simultaneously, called dual-task cost (DTC), can predict fall risk and cognitive decline. However, findings are mixed and predictive validity is modest. This study aims to define a neural marker of DTC that, when combined with a behavioral measure, could provide a more robust predictor. To this end, we acquired event-related potentials (ERP) in 36 healthy individuals aged 65 years and older who performed a cognitive Go/NoGo response inhibition task while sitting (i.e., single task) or walking (i.e., dual-task). To determine a neural marker of DTC, we considered differences in DTC between participants. Guided by findings in young adults showing a link between low behavioral DTC and reduction in NoGo-ERP amplitude during walking, we tested this prediction in individuals aged 65 years and older. Participants experienced dual-task cost when performing the Go/NoGo task while walking. They also exhibited a reduced ERP amplitude performing the Go/NoGo task during walking compared to sitting. When participants were split into two equal groups defined by their Go/NoGo performance, however, only the group with minimal DTC revealed a NoGo-ERP modulation during walking. Our data suggest that low DTC is linked to walking-related change in neurocognitive processing (i.e., NoGo-ERP amplitude reduction). The NoGo-ERP may serve as neural DTC marker to help improve early prediction of fall risk and cognitive decline in aging.

